# Neonatal Acute Kidney Injury—An Australian and New Zealand Perspective

**DOI:** 10.1111/jpc.70464

**Published:** 2026-06-09

**Authors:** Saumil Desai, Thomas Forbes, Simon A. Carter, Alison L. Kent, Chanel Prestidge, David T. Selewski, Eveline Staub, Anke Raaijmakers

**Affiliations:** ^1^ Newborn Emergency Transport Services, Perth Children's Hospital Nedlands Western Australia Australia; ^2^ Neonatology Clinical Care Unit, Perth Children's Hospital Nedlands Western Australia Australia; ^3^ Centre for Neonatal Research and Education, University of Western Australia Nedlands Western Australia Australia; ^4^ Murdoch Children's Research Institute Melbourne Australia; ^5^ Department of Nephrology Royal Children's Hospital Melbourne Australia; ^6^ Department of Paediatrics University of Melbourne Melbourne Australia; ^7^ Department of Pediatrics University of Rochester Rochester New York USA; ^8^ Department of Paediatric Nephrology Starship Children's Hospital Auckland New Zealand; ^9^ Department of Paediatrics University of Arkansas for Medica Sciences Little Rock Arkansas USA; ^10^ Department of Neonatology Royal North Shore Hospital St Leonards New South Wales Australia; ^11^ University of Sydney, Northern Clinical School, Royal North Shore Hospital St Leonards New South Wales Australia; ^12^ Paediatric Nephrology, Sydney Children's Hospital Randwick Australia; ^13^ School of Women's and Children's Health, University of New South Wales Sydney Australia

## Abstract

Acute kidney injury (AKI) is increasingly recognized as a critical and underdiagnosed condition among neonates, with significant short and long‐term implications for survival, kidney function, and long‐term cardiovascular health. Neonatal physiology, including ongoing nephrogenesis, immature renal haemodynamics, and limited glomerular filtration, makes this population vulnerable to AKI. Advances in consensus definitions, particularly the modified neonatal Kidney Disease Improving Global Outcomes (KDIGO) criteria, have enhanced epidemiologic understanding and facilitated global research collaboration. Despite this, significant variability in AKI surveillance, diagnosis, and follow‐up persists. Recent research efforts within Australia emphasize the need to incorporate AKI metrics into quality registries and improve equity of kidney care for infants. Integration of protocolized monitoring, preventative strategies such as nephrotoxin stewardship and methylxanthine therapy, and long‐term follow‐up for AKI survivors are essential to mitigating progression to chronic kidney disease. This narrative review synthesizes evolving evidence in neonatal renal physiology, AKI definition and biomarkers, epidemiology, management, and policy directions, emphasizing opportunities for education, protocol development, and collaborative improvement across Australia, New Zealand and beyond.

## Brief Key Points

- Acute Kidney Injury (AKI) is common among critically ill neonates and is directly linked with morbidity and mortality. As awareness and data collection improve, AKI is now recognised as a multisystem syndrome affecting overall survival and quality of life.
- Although standardized definitions and structured care protocols have improved AKI detection and outcomes, neonatal AKI still lacks universal diagnostic consistency, particularly across subspecialties in Australia and New Zealand.
- Diagnostic practices also remain inconsistent compared with adult and paediatric populations, underscoring the need for continued education, collaboration and implementation of long‐term follow‐up.

## 1 Introduction

Acute Kidney Injury (AKI) occurs commonly in critically ill neonates and is associated with increased morbidity and mortality [1, 2, 3, 4]. As the impact of AKI in neonates has become increasingly clear, global efforts have shifted towards education, highlighting its profound impact on both short‐ and long‐term outcomes. As data collection and awareness have improved, AKI is emerging as a multisystem syndrome with implications beyond the kidneys, affecting survival rates and quality of life [5, 6, 7, 8, 9, 10, 11, 12].

Advances in clinical practice and research have made AKI recognition, management, and prevention (in adults) central priorities for clinicians worldwide. The implementation of a standardized AKI definition and improving protocolization of care have led to more reliable detection, recognition, and improving outcomes [13, 14, 15]. Epidemiologic data show that the AKI burden in hospitals and intensive care units is high, with its downstream impact including chronic kidney disease (CKD), hypertension, and enhanced risk for systemic complications [7, 9, 11, 16, 17, 18]. AKI precipitates injuries beyond the kidney, with adverse effects on the lungs, brain, immune system, and overall homeostasis, reiterating the necessity for cross‐disciplinary care.

The neonatal population presents unique challenges and opportunities. Educational efforts to standardize surveillance and AKI diagnosis have led to increased recognition and decreased incidence of AKI [19]. These efforts have begun to ‘move the bar’ within Australia and New Zealand, bringing neonatal AKI out of obscurity and into routine interdisciplinary and neonatal intensive care [17]. Unlike in paediatric and adult settings, where diagnostic criteria have been widely embraced, perceptions and practices surrounding neonatal AKI continue to vary across subspecialties [20].

This international multidisciplinary state of the art review on neonatal AKI will highlight neonatal renal physiology, consensus AKI definitions and biomarkers, epidemiology with emphasis on Australian and New Zealand populations, multisystem impacts, surveillance protocols, treatment, prevention, and long‐term follow‐up recommendations.

## 2 Foetal and Neonatal Renal Physiology

Several aspects of renal development warrant special consideration in neonatal AKI. These include the duration of nephrogenesis, changes in renal blood flow, tubular immaturity, diuresis and the dynamic nature of neonatal glomerular filtration rate (GFR) [21, 22, 23, 24, 25, 26].

Nephrogenesis begins from the 5th week until ~34–36 weeks of gestation. Nephron endowment is highly variable (0.2–2.7 million nephrons per kidney) and preterm birth interrupts this process with lower endowment, predisposing to both AKI and CKD [7, 21, 22, 27, 28]. Studies show that accelerated nephrogenesis and abnormal glomerular architecture in preterm and/or intrauterine growth restricted (IUGR) neonates lead to poor kidney reserve and increased CKD risk later in life, potentially intensified by nephrotoxins or other kidney insults [16, 29, 30, 31]. Moreover, maternal kidney health has been linked with neonatal renal parenchymal thickness and potential nephron endowment and function, as well as congenital abnormalities of the kidney and urinary tract [32, 33, 34].

Postnatal adaptation of renal blood flow is a function of vascular resistance which decreases rapidly in the first days of life, with further reduction over the ensuing weeks until early childhood. The proportion of cardiac output directed to the kidneys increases accordingly, from 4% to 6% immediately after birth to 10%–15% at the end of the first week, and closer to the adult proportion of 25% after the neonatal period. Regulation of renal blood flow in neonates is largely dictated by the renin‐angiotensin system (RAS) and prostaglandins [35, 36]. These systems are crucial for maintaining GFR, renal perfusion, and systemic blood pressure, but in the neonatal period—especially in preterm infants—they are not fully developed or are significantly dysregulated [24]. The immature regulatory mechanisms make them susceptible to conditions common in sick newborns that alter perfusion (sepsis, nephrotoxins, etc.), leading to AKI [22, 29, 37].

Healthy neonates have an expected postpartum diuresis, due to limited urinary concentration ability and reduced GFR, which is more pronounced in those born preterm [25]. They have markedly reduced concentrating capacity (maximum urinary osmolality 200–600 mOsm/kg), impaired sodium handling, and a blunted response to fluid stress [2]. In term neonates the GFR is 10–20 mL/min/1.73 m^2^ and increases to 30–40 mL/min/1.73 m^2^ by the end of the second postnatal week [23, 26, 38]. These features underscore the narrow physiological reserve of the neonatal kidney and the importance of tailored fluid, haemodynamic and medication management.

## 3 Definition and Biomarkers

AKI research has been revolutionized by the adoption and utilization of a standardized definition leading to improved understanding of AKI epidemiology and outcomes. Initial staged definitions have been further refined and unified in the Kidney Disease Improving Global Outcome (KDIGO) classification [39, 40, 41].

Early work in neonatal AKI was hampered by the utilization of multiple varying definitions. In 2013, a neonatal modified KDIGO definition was proposed to be utilized in research and clinical care (Table 1) [13]. They incorporate a rise in serum creatinine and decreases in urine output, tailored to neonatal physiology [40].

**TABLE 1:** Neonatal KDIGO AKI definition (based on Zappitelli et al. [13] and KDIGO [40]).

Feature	Stage 1	Stage 2	Stage 3
Serum creatinine (SCr) change from baseline^a^	↑ ≥ 26.5 μmol/L (0.3 mg/dL) within 48 h or 1.5–1.9 × baseline^a^	2.0–2.9 × baseline^a^	≥ 3.0 × baseline^a^ or SCr ≥ 221 μmol/L (2.5 mg/dL) or initiation of renal replacement therapy
Urine output cut‐offs	< 0.5–1 mL/kg/h for 24 h	< 0.5 mL/kg/h for ≥ 12–24 h	< 0.3 mL/kg/h for ≥ 24 h (or anuria ≥ 12 h)

^a^ Previous lowest value.

Serum creatinine as a neonatal AKI biomarker is fraught with limitations. Neonatal creatinine initially reflects maternal levels, then undergoes a postnatal trajectory influenced by volume contraction, reduced GFR, and low muscle mass. Delayed rises mean that creatinine can lag true kidney injury by 48–72 h, and in very preterm infants, a delayed fall or transient rise may reflect physiological adaptation rather than structural injury [15]. Urine output in neonates remains a challenging aspect due to difficulties in routine and accurate measurement.

To improve early detection of AKI there has been a push to identify novel biomarkers that are true markers of injury. Urine neutrophil gelatinase‐associated lipocalin (uNGAL), which is shed in the renal tubules in response to injury, has been studied in older neonates and children. Neonates, however, have demonstrated significant variability in uNGAL in the absence of AKI [42], and further studies are required to determine optimal thresholds for AKI risk assessment. It is unlikely that any one test will be sufficiently sensitive to discriminate injury without risk prediction tools guiding their use [43]. Until such time as novel biomarkers reach clinical application in Australia and New Zealand, the improved surveillance of at‐risk babies using serum creatinine and urine output remains the most immediately impactful strategy (Table 1).

## 4 Epidemiology and Outcomes of Acute Kidney Injury in Neonates

### 4.1 General NICU Populations

The Assessment of Worldwide Acute Kidney Injury Epidemiology in Neonates (AWAKEN) study is the largest multicentre study of neonatal AKI to date, including 2022 critically ill neonates across 24 centres in four countries [1]. Using the neonatal KDIGO definition, AKI occurred in 30% of the cohort and was independently associated with increased mortality (adjusted OR 4.6) and prolonged length of stay (an adjusted increase of 8.8 days) [1]. A meta‐analysis of 201 studies confirmed these findings (98 228 neonates in 45 countries) reporting a pooled AKI incidence of 30% and an increased odds of mortality (OR 3.4; 2.9–4.0) [4]. AKI is also associated with higher healthcare costs. In a recent study, a diagnosis of neonatal AKI was associated with an adjusted cost increase of nearly 60 000 USD per admission [44]. A unique aspect to the care of critically ill neonates is their prolonged hospitalization, which exposes them to the possibility of recurrent episodes of AKI. In a secondary analysis of the AWAKEN study, Rutledge et al. found that 22% of those with AKI had a subsequent episode associated with increased length of stay [45].

### 4.2 Premature and High‐Risk Neonates

**FIGURE 1:**
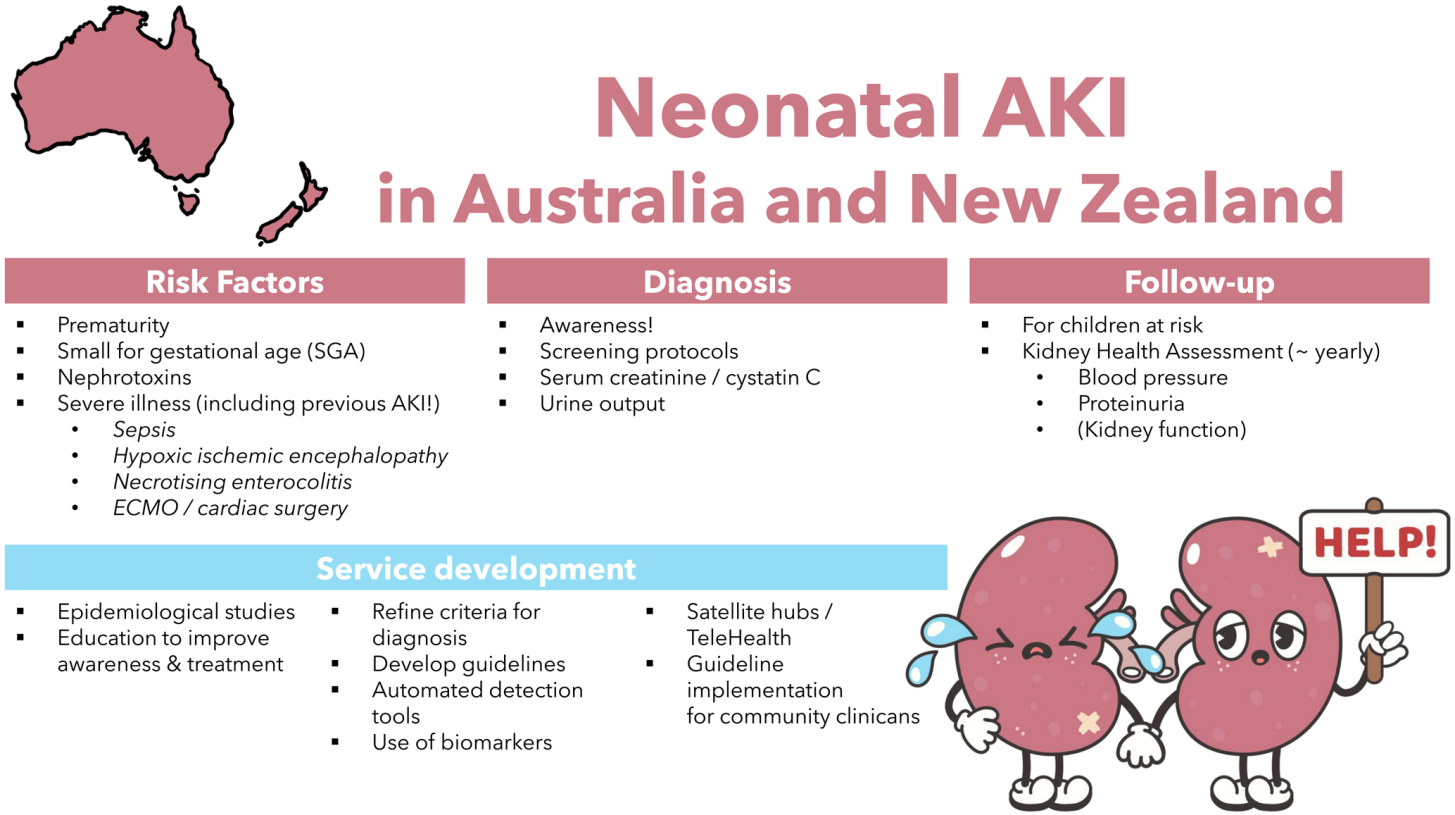
Neonatal AKI in Australia and New Zealand—awareness and service development.

AKI risk differs significantly by gestational age. The highest incidence of AKI in the AWAKEN study occurred in those less than 29 weeks gestational age (47.9%), followed by NICU‐admitted term infants. Those born at 29–36 weeks gestation had the lowest rates of AKI (18.3%), reflecting lower rates of critical illness when admitted to the NICU for feeding management (Figure 1).

AKI in neonates can occur more frequently in specific populations with unique pathophysiologic states including extracorporeal membrane oxygenation (ECMO), necrotizing enterocolitis (NEC), hypoxic ischemic encephalopathy (HIE), sepsis, patent ductus arteriosus etc. [46, 47, 48] For example, neonatal cardiac surgery is a significant risk factor for AKI and is performed only in select centres with routine peritoneal dialysis performed post‐operatively [49]. Moreover, neonates on ECMO have been shown to have rates of AKI as high as 50%–70% with associated adverse outcomes [50, 51, 52]. Similarly, NEC contributes to AKI through systemic inflammatory responses, sepsis, hypotension, and increased intra‐abdominal pressure further compromising renal perfusion and function [53].

In neonates with HIE, AKI risk is heightened due to systemic hypoxia and multi‐organ dysfunction, reflecting both direct kidney injury and secondary effects from supportive therapies [46]. AKI occurs in 40%–70% of neonates with HIE [11, 54, 55, 56, 57]. In neonates with HIE, AKI has been shown to be associated increased morbidity (length of stay, length of mechanical ventilation) and mortality. Furthermore, AKI during therapeutic hypothermia has been shown to be associated with increased risk of abnormal MRI at 7–10 days postnatally [11]. In a study of 101 neonates with HIE, AKI was associated with the composite long‐term outcome of death or long term disability [10].

PDA represents a common risk factor for AKI in neonates and presents one of the more challenging aspects of neonatal care. The risk of AKI in PDA is driven by physiological shunting of blood decreasing kidney perfusion. While the risk of AKI can be compounded by treatment with nephrotoxic medications (i.e., NSAIDS/COX inhibitors), the treatment effect may ultimately decrease the instance of moderate‐to‐severe AKI [58].

### 4.3 Acute Kidney Injury in Neonatal Cohorts in Australia and New Zealand

In Australia and New Zealand, care for high‐risk neonates is centralized in tertiary, metropolitan hospitals, whilst newborns in rural and remote populations face specific challenges. One quaternary NICU audit in Melbourne [59] (*n* = 2000) has found a low prevalence of preterm infants and a correspondingly low risk of AKI (10%) but a similar increase in mortality and length of stay compared with international literature. Surgery of any kind carried a strong association with AKI. Neonates with or at risk for AKI in rural and remote locations face a very separate set of issues with timely access to supportive care and risks associated with hospital transfer, whilst delays in laboratory testing may hamper early recognition of AKI.

A recent multicentre study in Australia and New Zealand (NeoKANZ) reported an AKI incidence of 26% [60]. Similar to the AWAKEN data, the moderately preterm group (29–35 weeks) were least affected, but the extremely preterm neonates < 29 weeks had lower incidence of AKI (30%). Other studies from Australia confirm that among preterm infants, those requiring resuscitation and endotracheal intubation at birth show higher subsequent AKI rates, emphasizing the role of early critical illness severity. Small for gestational age (SGA) status further exacerbates AKI risk. NeoKANZ and other ANZ cohorts highlight that the proportion of neonates affected by AKI exceeds that of several widely recognized complications such as NEC and intraventricular haemorrhage, yet AKI surveillance and documentation lag behind [1, 16, 20, 61, 62].

Aboriginal and Torres Strait Islander (ATSI) peoples face complex, multidimensional risks around neonatal kidney health and consequently their lifetime risk of kidney disease. These risks form an intergenerational cycle with major negative health consequences, both for kidney health and more broadly [63]. ATSI children and adults have a much higher burden of acute and chronic kidney disease, due to multiple risk factors including higher rates of post‐Streptococcal glomerulonephritis, hypertension and metabolic risk factors including diabetes and smoking [64, 65]. Women with CKD and kidney failure also have higher rates of pre‐eclampsia, premature delivery, intra‐uterine growth restriction and babies small for gestational age [66]. As discussed earlier, this leads to reduced nephron endowment for their children at birth with an increased lifetime risk of AKI and CKD, thus re‐establishing the same set of maternal risk factors [67, 68]. This cycle is then compounded by structural and culturally based inequity in access to care and results in the perpetuation of significant kidney health disparity. Any strategy to improve neonatal AKI outcomes in ANZ must therefore emphasize equity, culturally safe care, and early life kidney health in ATSI communities.

## 5 Surveillance

Protocolized surveillance is critical but remains underutilized in critically ill neonates [17]. Studies such as AWAKEN [1] and NeoKANZ [60] highlight the practice variation and relatively small proportion of neonates with protocol based creatinine monitoring, underscoring the need for systematic screening [1, 69]. Almost a quarter of the study population of NeoKANZ did not have enough data to ascertain AKI status (either by serum creatinine or urine output measurements), identifying a clear opportunity for improved surveillance.

The opportunities for improved education and surveillance were highlighted in an international survey of neonatologists and nephrologists performed in 2018 and replicated in 2026 [20, 70]. These studies identified gaps between neonatologists and nephrologists in the understanding of diagnosis, risks and outcomes associated with neonatal AKI [20, 70]. Furthermore, there was a lack of systematic utilization of standardized protocols for monitoring kidney function in neonates exposed to nephrotoxic medications such as aminoglycosides and indomethacin [20, 70].

Emerging data indicate that protocolized diagnosis leads to markedly improved outcomes in neonatal AKI [19, 71]. While centres use different educational efforts and protocols, the interventions showed improvements in neonatal AKI diagnosis, surveillance and follow‐up (Figure 1). Furthermore, these studies showed that efforts led to a decrease in the incidence of AKI [19]. This work highlights the need and impact of multidisciplinary efforts aimed at neonatal AKI education and protocolized surveillance.

## 6 Prevention and Treatment

Preventing neonatal AKI requires both reduction of modifiable risk factors and proactive surveillance of high‐risk infants. Structured surveillance programs such as Baby‐NINJA (Nephrotoxic Injury Negated by Just‐in‐Time Action [72]) have been successful at decreasing the incidence of nephrotoxic AKI. In this study, high‐risk neonates would be recommended daily serum creatinine checking until 2 days after the end of the exposure. The implementation of this protocol was shown to be feasible and led to decreased rates of nephrotoxic AKI. Such systematic monitoring not only provides opportunities for prompt intervention but also fosters a culture of vigilance, where prevention becomes the priority.

Pharmacological strategies have begun to show great promise in protecting neonatal renal function. Methylxanthines such as caffeine and theophylline have demonstrated renal‐protective effects due to their adenosine receptor antagonism [15, 73]. By improving renal blood flow, limiting ischemia–reperfusion damage, reducing oxidative stress, and enhancing urine output, these agents address multiple pathophysiological processes underlying neonatal AKI. Importantly, their dual benefit (stabilizing cardiorespiratory function while supporting kidney health) makes them uniquely valuable in high‐risk neonates. Growing evidence supports their potential role as both a therapeutic and preventive measure, offering solutions that can be integrated into existing clinical frameworks [74, 75].

## 7 Long‐Term Follow‐Up

Recent consensus recommendations emphasize lifelong follow‐up for survivors of neonatal AKI, with cardiovascular and kidney surveillance given equal importance, especially in former preterm neonates [17, 76]. Routine monitoring should extend beyond cardiac status to include kidney function, hypertension, and secondary complications (Figure 1). Further longitudinal protocolized follow‐up studies of these high‐risk neonatal populations are needed to allow for the early identification of those at risk for chronic kidney disease later in life.

Ten percent of infants in Australia and New Zealand are born preterm and will also require lifelong screening for abnormal kidney health. Therefore, the development of education, guidelines and referral pathways for community health services to perform this screening (including blood pressure measurement in infants) will be critical.

Despite these recommendations, implementing long‐term follow‐up presents significant challenges. AKI is frequently omitted from NICU discharge summaries, but simple quality improvement strategies within units can improve recognition of the need for follow up [69]. Guideline development and targeted education of community healthcare providers and families in Australia will be necessary to improve capacity to deliver follow up and support accurate blood pressure measurement in infancy (Figure 1).

## 8 Future Directions

At the heart of the improvement in our understanding of the incidence and impact of neonatal AKI has been multidisciplinary collaboration between neonatologists and nephrologists. This led to the formation of the Neonatal Kidney Collaborative in 2013, and the subsequent design and execution of the AWAKEN study in 2014. In 2024, the Australia and New Zealand Chapter of the NKC was founded as the first regional group to tailor educational, research and advocacy efforts to a local context very different to the USA, such as the geographical distribution of centres and specialists as well as limitations in access to highly specialized renal replacement therapy for neonates. In 2025, the Assessment of Worldwide Acute Kidney Injury Epidemiology of Neonates 2.0 (AWAKEN 2.0) study commenced to update and expand our understanding of neonatal AKI.

One of the most exciting technologic developments in neonatal critical care nephrology has been the development or adaptation of devices to provide continuous renal support therapy to even the smallest neonates. Prior to the development of such devices, CRRT in neonates was limited to the utilisation of adult devices with large extracorporeal volumes and degrees of precision that were not ideal for neonates. These devices have provided the opportunity to provide CRRT to neonates that previously would not have had any options. The continued deployment, optimisation, and utilisation of these devices will drive neonatal nephrology critical care going forward.

Australia and New Zealand NICUs contribute annual data of outcomes of premature and critically ill term neonates to a binational database—the Australian and New Zealand Neonatal Network (ANZNN). This data is published annually and allows units to monitor their performance and initiate clinical practice improvement programs to continually improve outcomes. Currently the ANZNN collects important morbidities but not AKI. The data collection also includes neurodevelopmental outcomes at 2–3 years of age, but no cardiac or kidney outcomes. The ANZ Chapter of the NKC is actively advocating for AKI and treatment with renal replacement therapy to be included in these reported outcomes. Support for clinical practice improvement programs to reduce nephrotoxic AKI, improve diagnosis and reporting of AKI, establish guidelines for caffeine administration in neonates being cooled for HIE and guidelines for long‐term follow‐up are urgently needed to improve long term kidney health of NICU graduates in Australia and New Zealand (Figure 1).

## 9 Conclusions

Neonatal AKI represents one of the most rapidly growing fields of clinical research across neonatology. AKI is common in this population, and the deleterious impact is clear. Multidisciplinary collaboration is at the heart of improving outcomes in this vulnerable population. Ongoing advances in education, guideline development for prevention and surveillance, and collection of kidney outcomes will improve long‐term kidney health in this vulnerable population. Continued research and clinical collaboration across disciplines can tangibly improve neonatal AKI outcomes. Future research directions must include the utilization of novel biomarkers and the deployment and optimization of neonatal RRT devices.

## Funding

D.T.S. is supported, in part, by the Arkansas Children's Research Institute and the Arkansas Biosciences Institute. D.T.S. is on the speaker's bureau for Bioporto. D.T.S. is an author for Up‐To‐Date.

## Ethics Statement

The authors have nothing to report.

## Consent

The authors have nothing to report.

## Conflicts of Interest

The authors declare no conflicts of interest.

## Data Availability

Data sharing not applicable to this article as no datasets were generated or analysed during the current study.
